# IL-27 regulates the adherence, proliferation, and migration of MSCs and enhances their regulatory effects on Th1 and Th2 subset generations

**DOI:** 10.1007/s12026-017-8929-8

**Published:** 2017-06-14

**Authors:** Fenghuang Xu, Junzhu Yi, Zhuoya Wang, Yejia Hu, Chunlei Han, Qun Xue, Xueguang Zhang, Xiying Luan

**Affiliations:** 1 Department of Immunology, Binzhou Medical University, Yantai, Shandong Province 264003 People’s Republic of China; 2 Department of Pathophysiology, Binzhou Medical University, Yantai, Shandong Province 264003 People’s Republic of China; 3 Department of Health Statistics, Binzhou Medical University, Yantai, Shandong Province 264003 People’s Republic of China; 4 Medical College of Soochow University, Suzhou, Jiangsu 215006 People’s Republic of China; 5 Taishan Scholar Immunology Program, Binzhou Medical University, Yantai, Shandong Province 264003 People’s Republic of China

**Keywords:** IL-27, Mesenchymal stromal cells (MSCs), Th1, Th2, IL-10, Programmed death ligand 1 (PDL1)

## Abstract

Interleukin 27 (IL-27) regulates T cell function and is involved in inflammation. It has been reported that human placenta-derived mesenchymal stromal cells (hPMSCs) can inhibit T cell responses and attenuate inflammation reactions. However, it is unclear whether IL-27 can regulate hPMSC function. Here, we examined the effects of IL-27 upon adherence, migration, and proliferation as well as the immunomodulatory effects of hPMSCs. The results show that IL-27 receptor α chain (IL-27Rα) is expressed in hPMSCs. IL-27 at 30 ng/ml inhibited hPMSC adherence and proliferation, while the migration of hPMSCs was promoted with IL-27 at doses of 20 or 30 ng/ml, as determined with use of real-time cell analysis (RTCA). Moreover, IL-27 promoted regulatory effects of hPMSCs through enhancing Th2 and suppressing Th1 subset generation from activated T cells in human peripheral blood. IL-27 also enhanced the ability of hPMSCs to secrete IL-10 from CD4^+^T cells through increased expression levels of the programmed death ligand 1 (PDL1) in hPMSCs via the Janus kinase (JAK)/signal transducer and activator of transcription 1 (STAT1) signaling pathway. In conclusion, IL-27 has significant modulatory effects on adherence, proliferation, and migration of hPMSCs. IL-27 increased PDL1 expression levels in hPMSCs via the JAK/STAT1 pathway, which then enhanced the regulatory effects of hPMSCs upon Th1 and Th2 cell generations and IL-10 secretion from CD4^+^T cells.

## Introduction

Interleukin 27 (IL-27) is a type-I-cytokine of the IL-12 cytokine superfamily and is predominantly secreted by macrophages and dendritic cells. The receptor for IL-27, which is expressed in T and B lymphocytes, is composed of a gp130 and IL-27 receptor α chain (the IL-27Rα is also known as WSX1 or TCCR) [1–3]. IL-27Rα and gp130 are expressed in human placenta tissue and bone marrow-derived mesenchymal stromal cells [4, 5]; however, little information is available regarding the expression of IL-27Rα in human placenta-derived mesenchymal stromal cells (hPMSCs). IL-27 has been shown to be involved with regulating the generation of T cell subsets. For example, IL-27, which can act as a pro-inflammatory cytokine to promote T-bet activation, then induces IL-12Rβ2 expression in T cells. Moreover, IL-27 can stimulate the differentiation of T cells into its subset, Th1, which can then promote the progress of inflammation [6]. IL-27 plays a pro-inflammatory role in graft versus host disease (GVHD) biology, as blocking of IL-27 signaling reduced GVHD in mice through augmenting Treg reconstitution [7]. IL-27 can also exert anti-inflammatory effects, through inhibiting the generation of T cells into its subset Th17 via signal transducer and activator of transcription 1 (STAT1) and STAT3 signaling pathways, thus alleviating the symptoms of experimental autoimmune encephalomyelitis (EAE) [1, 8]. Collating the information from these studies leads to the conclusion that IL-27 regulates immune function in infectious and autoimmune diseases via intervening with T cell subset transformation [9].

Results from numerous studies have demonstrated that mesenchymal stromal cells (MSCs) can be used in the treatment of inflammatory and autoimmune diseases [10]. Following tissue damage, MSCs migrate directly to damaged areas, where they regulate the activity and function of various immune cells to gradually reduce tissue damage and stimulate the secretion of growth factors which promote tissue repair by enhancing the differentiation of tissue precursor cells [11]. Moreover, inflammatory factors located at the sites of damage, such as interferon (IFN)-γ and tumor necrosis factor (TNF)-α, can, in turn, influence the biological function of MSCs. Zhang et al. reported that long-term IFN-γ could inhibit MSC proliferation [12]; and increased TNF-α secretion in systemic lupus erythematosus (SLE) serum significantly inhibited the migration and in vivo homing capacities of SLE bone marrow-derived MSCs (BMSCs) [13]. However, it is not clear whether IL-27 in these inflammatory reactions affects the activities of MSCs by regulating their proliferation, migration, and/or other biological functions of MSCs.

The immunomodulatory effects of MSCs on T cells have been observed in several paradigms. For example, MSCs can inhibit T cell proliferation and secretion of the Th1 cytokine IFN-γ, as well as induce the generation of the T cell subset CD4^+^CD25^+^Foxp3^+^Treg; however, the underlying mechanisms of these processes are not known [14, 15]. In addition, cytokines produced by activated T cells can also regulate MSCs [16]. Previous work from our laboratory has revealed that IFN-γ and TNF-α enhance the capacity for hPMSCs to induce the differentiation of activated T cells to IL-10^+^T cell subsets and, in this way, exert a remodeling effect upon activated T cells [17]. Moreover, IFN-γ can enhance MSCs ability to inhibit T cell proliferation by upregulating the expression of the programmed death ligand 1 (PDL1) in MSCs [18–20]. However, it is unclear whether IL-27 can affect T cell differentiation into Th1 or Th2 subsets by regulating PDL1 expression in hPMSCs.

In the current study, we first investigated the expression of IL-27Rα in hPMSCs, and then investigated the effects of IL-27 on adherence, migration, and proliferation of hPMSCs, as well as on the regulatory effects of hPMSCs on Th1 or Th2 cell generation. The significance of this study is its potential to identify the benefits of hPMSCs for using in clinical cell therapy.

## Materials and methods

### Isolation of hPMSCs

The hPMSCs used in this study have been isolated as described previously [21]. Briefly, placenta were obtained from healthy women who delivered after a full-term pregnancy. After the decidua tissue on placenta was removed and placenta was washed in D-Hanks solution, they were sectioned into small fragments and homogenized. The tissues were then incubated with 0.1% IV collagenase (Gibco, CA, USA) for 30 min at 37 °C, followed by filtering through a screen filter (100 mesh)**.** After centrifugation at 524×*g* for 10 min, cells were washed with D-Hanks solution, counted, and then incubated at 37 °C in a 5% CO_2_ environment. The cells were passaged once every 7–8 days with half of the medium replaced with new medium on day 3. The hPMSCs were identified by the following: (1) cell morphology as observed using microscopy, (2) the detection of cell surface antigens (CD105, CD73, CD90, CD34, CD14, CD19, and human leukocyte antigen-antigen D related (HLA-DR)) as determined using flow cytometry (FCM), and (3) the ability to differentially detect between bone and fat cells. Identified hPMSCs were used in the experiment after three passages. The project was approved by the Ethics Committee of the Affiliated Hospital of Binzhou Medical College, Yantai, and informed consent was obtained from all sample donors.

### Adipogenic and osteogenic inductions

HPMSCs were seeded in six plates for adipogenic and osteogenic induction. The hPMSCs reached 70% and 100% confluency for adipogenic and osteogenic induction, respectively. The medium was removed and then cultured with adipogenic and osteogenic differentiation medium. All differentiation processes were in strict accordance with the kit instructions (Wei Tong Biotechnology, China). Cells cultured without adipogenic or osteogenic differentiation medium were used as negative controls for adipogenic and osteogenic differentiation. For adipogenic staining, cells cultured with or without adipogenic differentiation medium were stained with Oil Red O after 14 days. For osteocyte staining, cells cultured with or without osteogenic differentiation medium were stained with Alizarin Red after 28 days.

### PBMC isolation

Peripheral blood mononuclear cells (PBMCs) were isolated from whole blood as described previously [22]. Briefly, the blood was obtained from healthy adults at the Central Blood Bank in Yantai City. Informed consent was acquired from all donors. After being anti-coagulated and diluted with an equal volume of D-Hanks solution, the blood samples were added to Ficoll separating medium. The PBMC suspension was prepared using a density gradient centrifugation method.

### RT-PCR analysis

Using CD3^+^T cells, which expressed IL-27Rα and served as a positive control and LNCaP cells as a negative control [23, 24], the messenger RNA (mRNA) expression of IL-27Rα in hPMSCs was detected using RT-PCR. Total RNA was extracted using TRIzol (Invitrogen, CA, USA). RNA was then transcribed into complementary DNA (cDNA) using the Revert Aid First Strand cDNA Synthesis Kit (Thermo Scientific, CA, USA) according to the operating instructions. PCR reactions were conducted using the 2 × Taq PCR Master Mix Kit (Thermo Scientific, CA, USA). The primer sequences were as follows: IL-27Rα—5′-ACC CAA ATG AAG CCA AAC GC-3′, 5′-CGC CCC ACA AAT CCT CTT CT-3′; β-actin—5′-GGC ACC CAG CAC AAT GAA-3′, 5′-GGA AGG TGG ACA GCG AGG -3′. PCR reaction conditions included 30 cycles at 94 °C for 2 min, 94 °C for 30 s, 55 °C for 30 s, and 72 °C for 1 min, followed by 72 °C for 5 min. PCR products were analyzed using 1% agarose gel electrophoresis.

### Gene sequencing

Gene sequencing was conducted for IL-27Rα mRNA in hPMSCs. A portion of the PCR products, as generated using procedures described above, was gene sequenced at the Shanghai Meiji Biomedical Co., Ltd. The gene sequencing results were compared with those of the National Center for Biotechnology Information (NCBI) using the Blast program (http: //www.ncbi.nlm.nih Gov/BLAST).

### Western blot analysis

Protein levels of IL-27Rα in hPMSCs were determined using Western blot with CD3^+^T cells serving as a positive control. Expressions of IL-27Rα in hPMSCs as determined on different culture days for one generation were then analyzed by Western blot, as were levels of phosphorylated STAT1 (P-STAT1) and STAT1 in these hPMSCs. HPMSCs were pretreated with the Janus kinase 1/2 (JAK1/2) inhibitor INCB018424 (20 ng/ml, Selleck, Shanghai, China) for 1 h before stimulation with IL-27 and incubated in the presence or absence of INCB018424 for an additional 1 h; P-STAT1 and STAT1 expressions were then measured by means of Western blot. After adding RIPA lysis buffer to hPMSCs, the cells were lysed on ice for 40 min, centrifuged, subjected to SDS-PAGE electrophoresis, and then transferred to PVDF membranes. Rabbit anti-human IL-27Rα (Bioss, Beijing, China), β-actin (Bioworld, Nanjing, China), phosphorylated STAT1 (Abcam, Cambridge, UK), or STAT1 (Proteintech, Wuhan, China) antibodies were incubated with membranes at 4 °C overnight. Secondary goat anti-rabbit antibody (1: 1000) (Santa Cruz, CA, USA) was added on the following day. Blots were further washed and developed with enhanced chemiluminescent substrate (Beyotime, Shanghai, China), and protein bands were then visualized using a Western blot imager.

### hPMSC adherence and proliferation

The hPMSC adherence and proliferation were analyzed with real-time cell analysis (RTCA) using the xCELLigence system E-Plate. Dulbecco’s modified essential medium (DMEM) supplemented with 10% fetal bovine serum (FBS) was added to the E-Plate, and the background cell index (CI) values were then recorded. The hPMSC suspension (10,000/well) was then added to the medium followed by IL-27 (Peprotech, NJ, USA) with final concentrations of 0, 10, 20, or 30 ng/ml. The hPMSCs were then incubated, and cell adherence and proliferation were observed at 5 min intervals using the RTCA analyzer. CI values were recorded, and adherence and proliferation were observed continuously for 6 and 80 h, respectively. The rate of cell growth was calculated based on the slope of the line between two given time points [25].

### hPMSC migration

RTCA was used to monitor the migration of hPMSCs in real-time as determined with use of the xCELLigence system CIM-plates. Medium containing 10% FBS (165 μl/well) was added to the lower chamber of the CIM-plates, while 30 μl/well DMEM was added to the upper chamber. After incubation for 1 h, baseline background levels were recorded. The hPMSC (40,000/well) suspensions without FBS were added to the upper chamber, followed by IL-27 at final concentrations of 0, 10, 20, or 30 ng/ml. RTCA was recorded at 5 min intervals. CI values and real-time dynamic analysis of migration were recorded over a 24 h period.

### Co-culturing hPMSCs and PBMCs

The 24-well plates containing hPMSCs (5 × 10^4^ cells/well) were treated with IL-27 (20 ng/ml) for 24 h. Then, PBMC (5 × 10^5^ cells/well) and PHA (10 μg/ml) (Solarbio, Beijing, China) were added [26]. PBMC and PBMC with PHA were used as negative and positive controls, respectively. After incubation for 72 h, PBMCs were analyzed using FCM analysis.

### PDL1 mAb blocking test

After incubation for 24 h, mouse anti-human PDL1 monoclonal antibody (mAb) (5 μg/ml) (BioLegend, CA, USA) was added to hPMSCs [26], followed by PBMC and PHA. PBMCs were then collected 72 h later and analyzed with use of FCM.

### Flow cytometry analysis

FCM was used to assess the regulatory effects of IL-27 on PDL1 expression in hPMSCs. The hPMSCs were cultured in 24-well plates for 24 h followed by the addition of varying concentrations of IL-27 (0, 10, 20, or 30 ng/ml). The hPMSCs were collected at 12, 24, 48, or 72 h post-IL-27 treatment. Phycoerythrin (PE)-labeled mouse anti-human PDL1 mAb (Ebioscience, CA, USA) were then added and incubated under dark conditions at 4 °C for 30 min. FCM analysis was then performed.

Membrane and intracytoplasmic molecules in T cells were detected by FCM. For intracellular staining, PBMCs were fixed, permeabilized, and stained with fluorescein isothiocyanate (FITC)-labeled mouse anti-human IFN-γ mAb, PE-labeled mouse anti-human IL-4 mAb, and IL-10 mAb. T cell surface molecules were stained with allophycocyanin (APC)-labeled mouse anti-human CD3 mAb, peridiinin-chorophyll protein complex (PerCP)-labeled mouse anti-human CD4 mAb (Miltenyi Biotec, Bergisch Gladbach, Germany). HPMSC surface molecules were stained with FITC-labeled mouse anti-human CD14 mAb, CD34 mAb, CD90 mAb, CD105 mAb, HLA-DR mAb, and PE-labeled mouse anti-human CD19 mAb, CD73 mAb (Miltenyi Biotec, Bergisch Gladbach, Germany), IL-27Rα mAb (R&D Systems, USA) at 4 °C for 30 min, then washed and analyzed with FCM. As negative controls for the fluorescent cell labeling, we employed appropriate isotype controls for the antibodies employed (Miltenyi Biotec, Bergisch Gladbach, Germany). All mAbs used were at concentrations recommended by the manufacturer.

### Statistical analysis

Data were expressed as mean ± standard deviation. All data were analyzed with SPSS20 statistical software. Differences among groups were calculated using ANOVA, and multiple comparisons were performed using the Bonferroni correction. A *P* < 0.05 was required for results to be considered statistically significant.

## Results

### Phenotypic characteristics and differentiation of hPMSCs

**Fig. 1:**
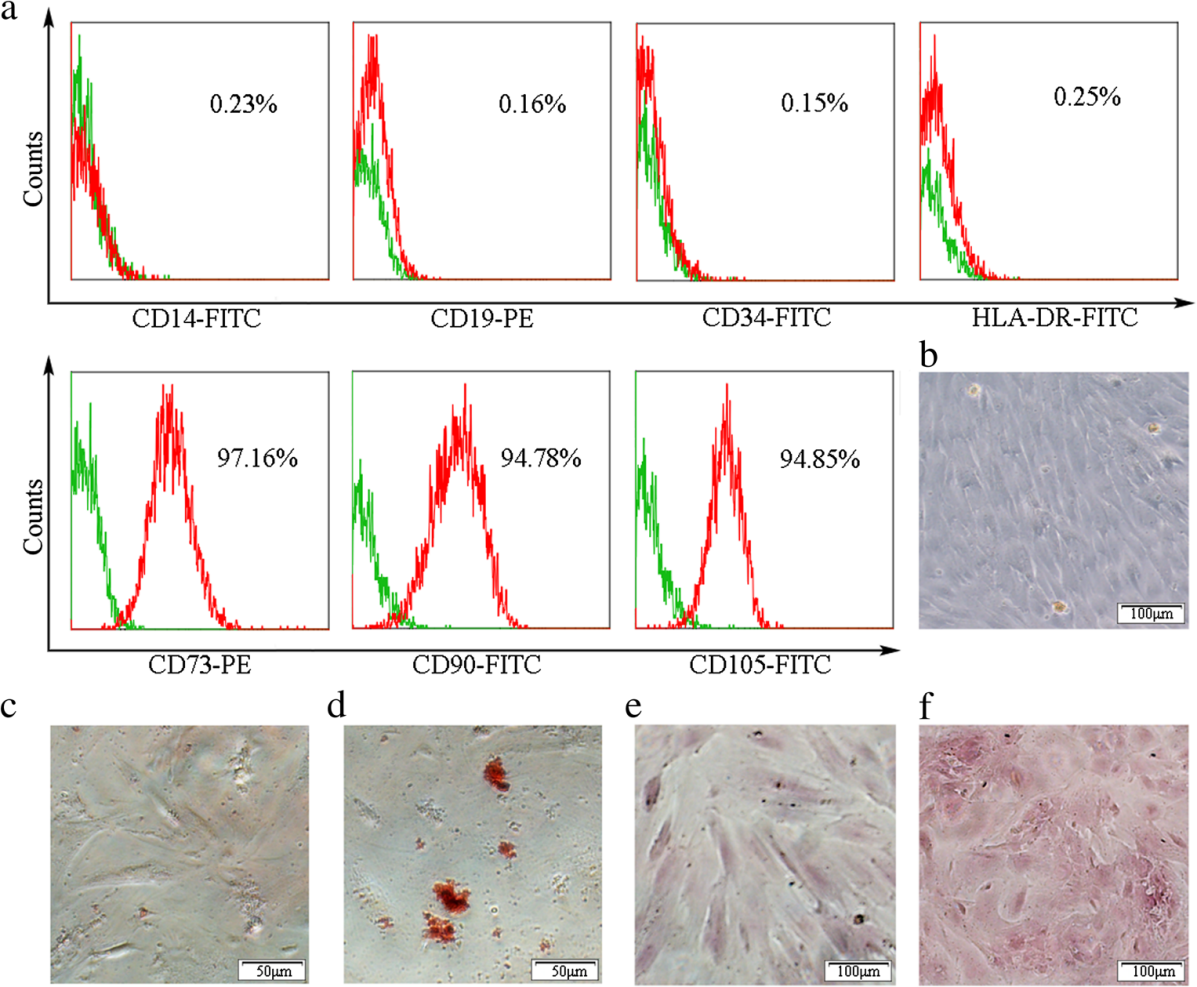
Immunophenotyping and differentiating capabilities of hPMSCs. Phenotypic characterization of hPMSCs by FCM analysis (**a**). Isotype controls are presented as *green* histograms. The *red line* in each histogram represents the specific expression of the indicated cell surface marker. The morphology of hPMSCs from three passages (**b**). Multilineage differentiation potential was assessed by their capacity to differentiate into adipocytes as stained by Oil Red O (**d**) and osteoblasts as stained by Alizarin red staining (**f**). Oil Red O (**c**) and Alizarin red (**e**) staining of hPMSCs as control (color figure online)

To verify that the cells separated and cultured were hPMSCs, the phenotype of hPMSCs was determined with use of FCM. The results showed that about 95% of the isolated hPMSCs expressed CD73 and CD90 and CD105, but not CD14, CD19, CD34, or HLA-DR (Fig. 1a). The hPMSCs cultured showed a fibroblast-like appearance and a capacity for attachment to plastic (Fig. 1b).

To determine whether hPMSCs could differentiate into multiple mesenchymal cell lineages, hPMSCs were cultured in adipogenic and osteogenic medium, respectively. In the adipocyte induction medium, adipocytes were verified by the presence of Oil Red O staining of fat globules (Fig. 1d). In the osteoblast induction medium, Alizarin red staining showed intracellular calcium deposits (Fig. 1f). As no fat globules and intracellular calcium deposits were found in hPMSC control groups which were cultured with DMEM supplemented with 10% FBS, these results indicated that the cells were, in fact, hPMSCs (Fig. 1c, e).

### Expression of IL-27Rα in hPMSCs

**Fig. 2:**
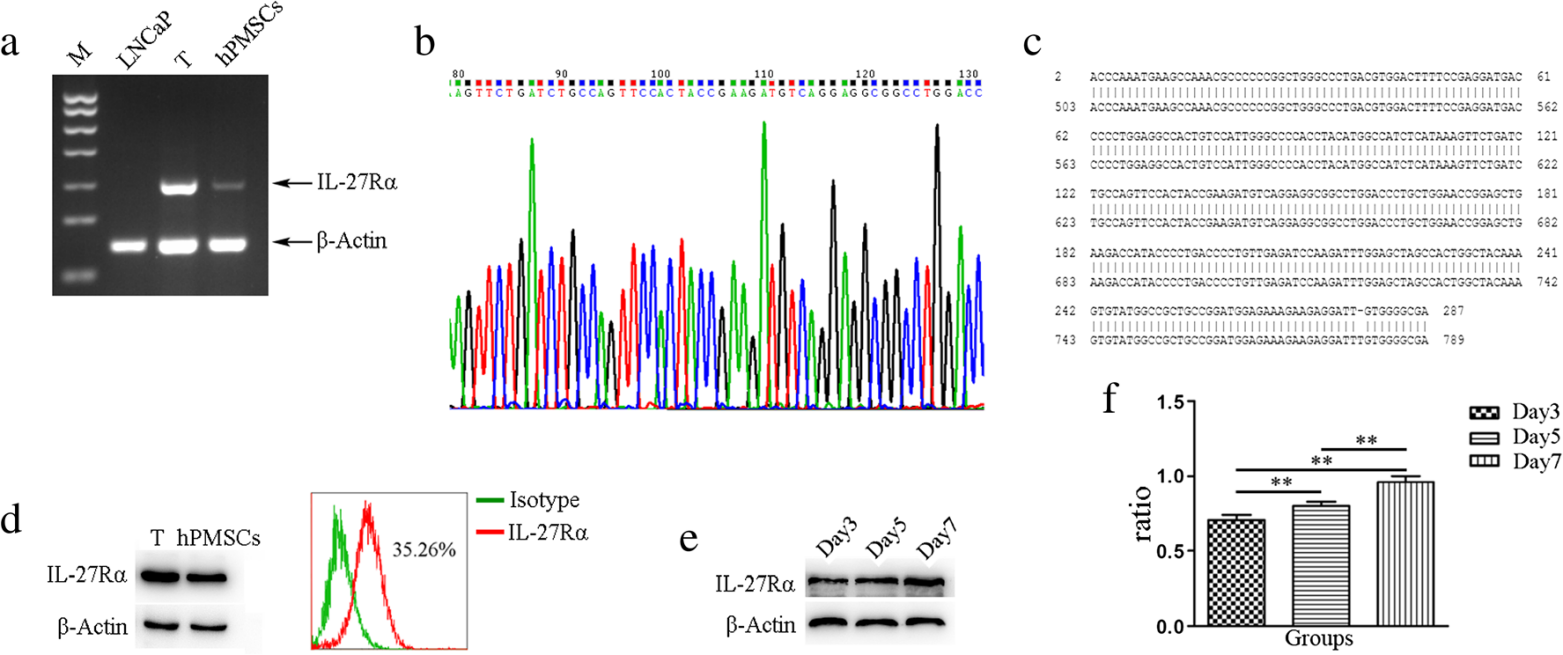
IL-27Rα expression in hPMSCs. **a** Detection of IL-27Rα mRNA expression in *hPMSCs* using RT-PCR; CD3^+^T cells were used as a positive control and *LNCaP* cells were used as a negative control. **b**, **c** Expression of IL-27Rα mRNA expression in hPMSCs using gene sequencing. **d** Detection of IL-27Rα protein expression in hPMSCs using Western blot and flow cytometry analyses. **e**, **f** Expression of IL-27Rα as determined at different time points(3, 5, and 7 days) from three passages of hPMSCs using Western blot

We first found that hPMSCs expressed IL-27Rα in gene and protein levels. IL-27Rα mRNA was expressed in hPMSCs as revealed with use of RT-PCR (Fig. 2a). To further corroborate these RT-PCR results, IL-27Rα PCR products were sequenced (Fig. 2b) and found to match with that of the 503–789 bp of IL-27Rα in the NCBI gene bank (Fig. 2c). Results obtained from Western blot and FCM also demonstrated that IL-27Rα protein was expressed in hPMSCs (Fig. 2d). As shown in Fig. 2e, levels of IL-27Rα in hPMSCs were not the same in different culture times, with maximal expressions of IL-27Rα being obtained on the seventh day of culture in passage three (*P* < 0.01; Fig. 2e, f).

### IL-27 regulates the adherence, migration, and proliferation of hPMSCs

**Fig. 3:**
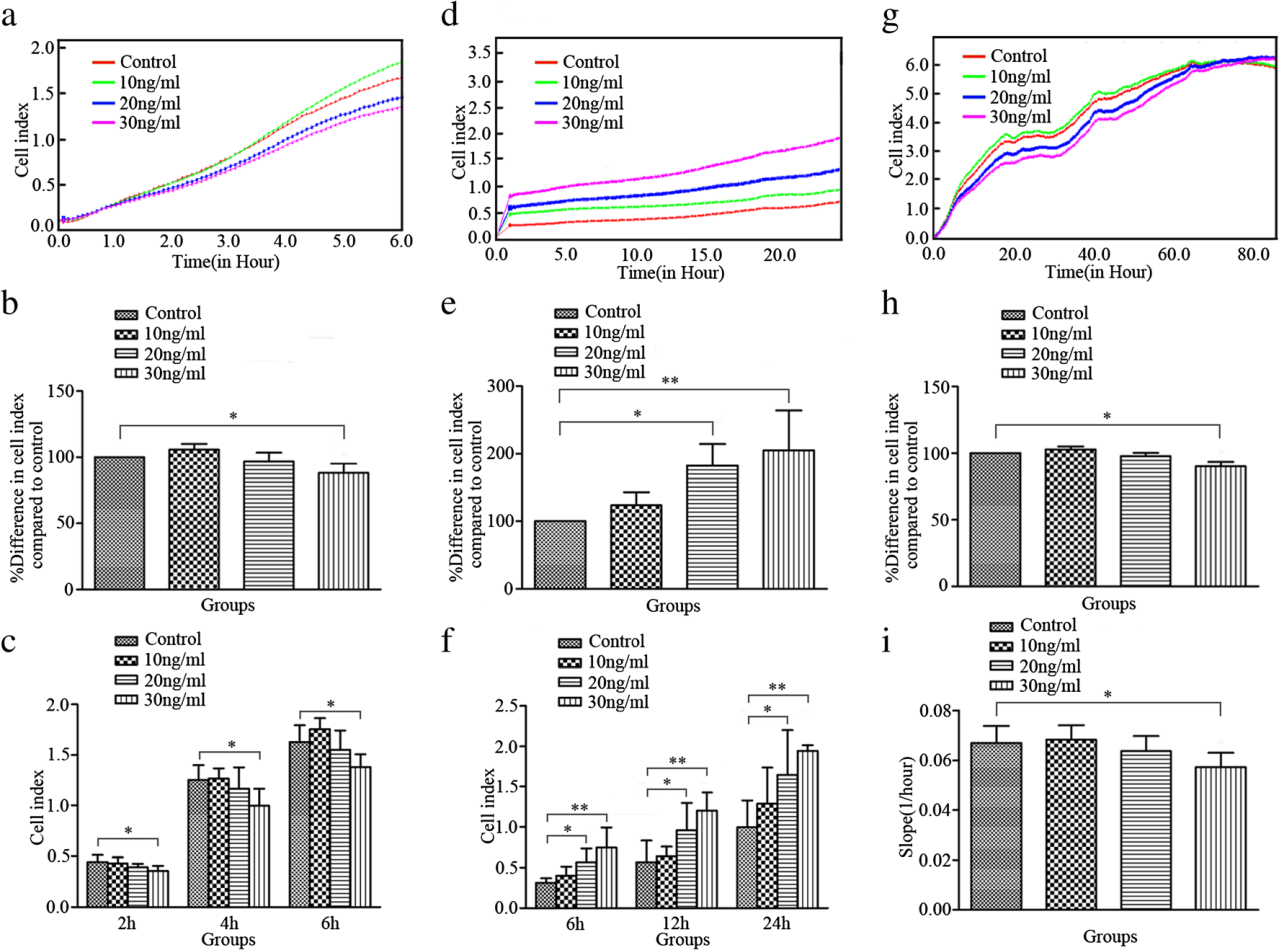
Effects of IL-27 on adherence, migration and proliferation of hPMSCs. Representative figure of CI values for adherence (**a**), migration (**d**), and proliferation (**g**) of hPMSCs treated with/without IL-27. Comparisons of the percent differences in the mean CI for adherence (**b**), migration (**e**), and proliferation (**h**) of hPMSCs treated with/without varying concentrations of IL-27. Representative figure on adherence (**c**) and migration (**f**) CI values for hPMSCs at different time points following treatment with the indicated concentrations of IL-27. The rate of proliferation (**i**) was measured by analyzing the slope of the line between 0 and 80 h after IL-27 treatment or not in hPMSCs. The results presented are from three independent experiments (*P < 0.05, **P < 0.01)

On the basis of above results, we next investigated the effects of IL-27 on hPMSC adherence, migration, and proliferation. Our results indicated that IL-27 can regulate the adherence, migration, and proliferation of hPMSCs (Fig. 3). IL-27 at 30 ng/ml inhibited hPMSC adherence (*P* < 0.05; Fig. 3a, b) as compared to controls, and incubation with 30 ng/ml of IL-27 for 2, 4, or 6 h resulted in significantly lower adherent CI values as compared to that of controls (*P* < 0.05; Fig. 3c). IL-27, at concentrations of 20 and 30 ng/ml, promoted hPMSC migration (*P* < 0.05; *P* < 0.01; Fig. 3d, e). Significant increases in CI values for hPMSC migration as compared to controls were observed when IL-27 (20 or 30 ng/ml) was incubated for 6, 12, or 24 h (*P* < 0.05; *P* < 0.01; Fig. 3f). Proliferation of hPMSCs was significantly inhibited by IL-27 at 30 ng/ml, but not at 10 or 20 ng/ml as indicated by CI values obtained within the 80 h of testing and changes in the slope of the curve between the 0 and 80 h periods (*P* < 0.05; Fig. 3g–i).

### IL-27 enhanced the regulation of hPMSCs on inducing the generation of Th1 and Th2 subsets from activated PBMCs

**Fig. 4:**
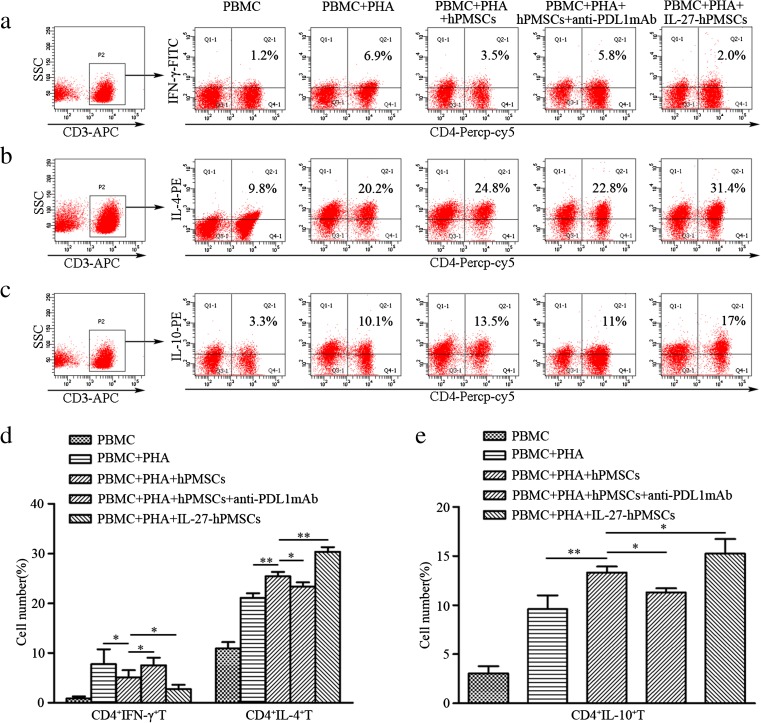
IL-27 enhanced the regulatory effects of hPMSCs on the generation of Th1 and Th2 cells from activated T cells and induced IL-10 secretion from CD4^+^T cells. PHA-activated PBMCs and hPMSCs were co-cultured with or without PDL1 blocking antibodies. Th1 (CD4^+^IFN-γ^+^T), Th2 (CD4^+^IL-4^+^T) cell subsets, and IL-10 secreting CD4^+^T cells were measured with use of FCM. **a**–**c** FCM figure for Th1 and Th2 cell subsets and IL-10 secreting CD4^+^T cells. **d**, **e** Th1 and Th2 cell subsets and secretion of IL-10 from CD4^+^T cells (mean ± SD). The results presented are from three independent experiments (*P < 0.05, **P < 0.01)

Results from FCM analysis indicated that hPMSCs promoted the differentiation of CD4^+^T cells into Th2 subsets (*P* < 0.01; Fig. 4b, d) while inhibiting the differentiation into Th1 subsets (*P* < 0.05; Fig. 4a, d), as compared to PHA activated T cells. Moreover, these regulatory effects of hPMSCs on Th1 and Th2 subset generations were enhanced after hPMSCs were pretreated with IL-27 (Th1: *P* < 0.05, Th2: *P* < 0.01; Fig. 4a, b, d). Our results also showed that PDL1 can significantly enhance hPMSCs’ regulatory effects on Th1 (*P* < 0.05) and Th2 (*P* < 0.05; Fig. 4a, b, d) subset generations.

### IL-27 upregulated the capacity for hPMSCs to induce IL-10 from CD4^+^T cells

Findings from our FCM analysis revealed that hPMSCs could induce secretion of IL-10 from PHA-activated CD4^+^T cells (*P* < 0.01; Fig. 4c, e). In response to IL-27 stimulation, hPMSCs induced significantly increased IL-10 secretion from CD4^+^T cells (*P* < 0.05; Fig. 4c, e). However, when the PDL1 blocking antibody was combined with hPMSCs, IL-10 secretion from CD4^+^T cells was significantly decreased (*P* < 0.05; Fig. 4c, e).

### IL-27 upregulated PDL1 expression in hPMSCs

**Fig. 5:**
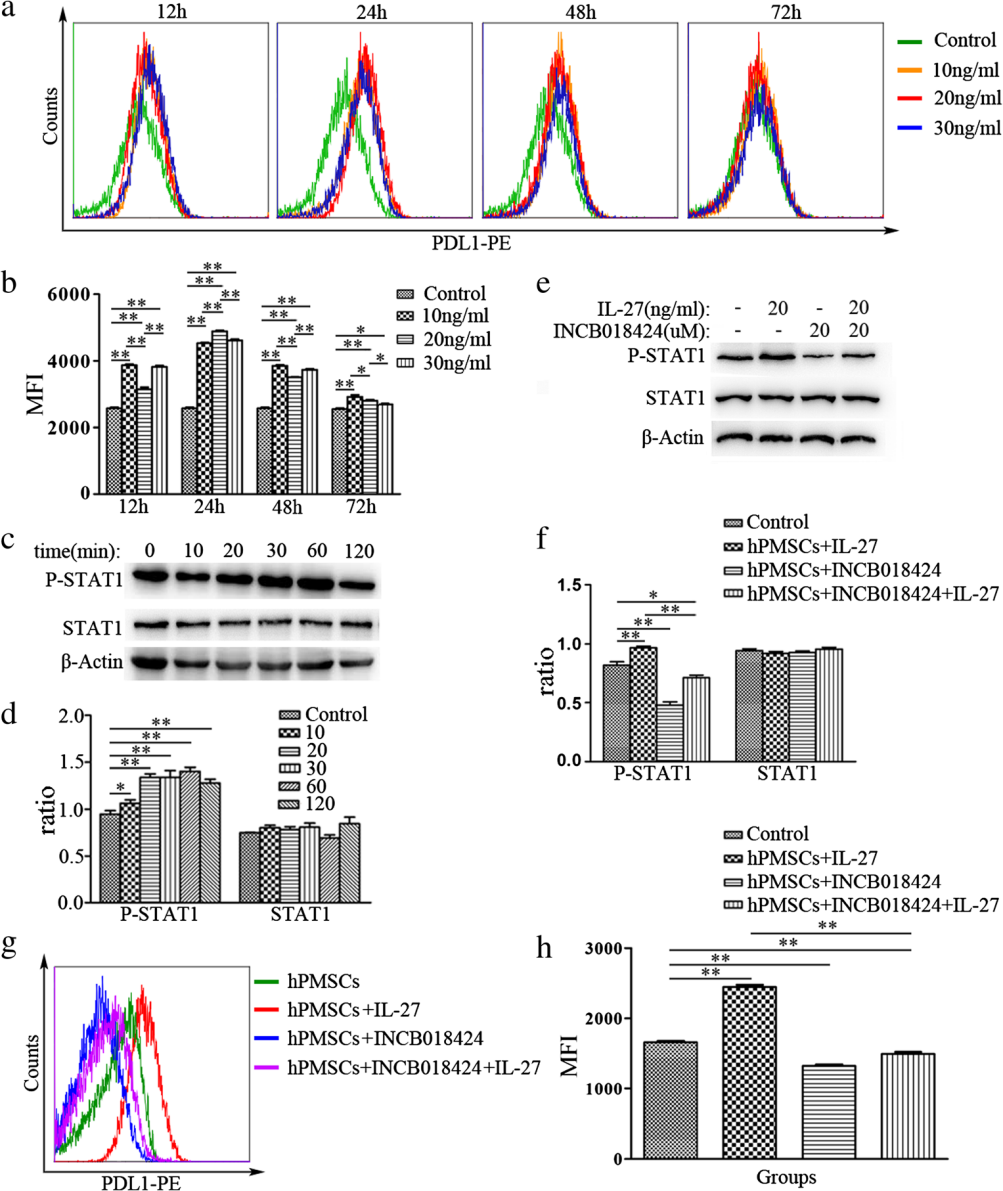
IL-27 upregulated PDL1 expression in hPMSCs via the JAK/STAT1 pathway. **a**, **b** IL-27 upregulated PDL1 expression in hPMSCs. **c**, **d** Detection of P-STAT1 and STAT1 expressions in IL-27 (20 ng/ml)-treated hPMSCs for 0, 10, 20, 30, 60, or 120 min as determined with use of Western blot. **e**, **f** P-STAT1 and STAT1 protein levels in IL-27-treated hPMSCs for 1 h, pretreated with/without the JAK1/2 inhibitor (INCB018424) for 1 h. **g**, **h** Expression of PDL1 in IL-27-treated hPMSCs for 24 h, which were pretreated with/without the JAK1/2 inhibitor (INCB018424) using FCM analysis. The results presented are from three independent experiments (*P < 0.05, **P < 0.01)

Results from a previous study in our laboratory revealed that PDL1 regulated the immunosuppression of hPMSCs on T cells [21]. In this study, PDL1 expression levels in hPMSCs were measured at 12, 24, 48 or 72 h after incubation with IL-27 at doses of 0, 10, 20, or 30 ng/ml. For all concentrations tested, IL-27 upregulated PDL1 expression in hPMSCs as compared with the 0 ng/ml group. In particular, hPMSCs incubated with IL-27 at 20 ng/ml for 24 h induced maximal levels of PDL1 expression (Fig. 5a, b). Further analysis revealed enhanced expression levels of P-STAT1 in hPMSCs after stimulation with IL-27 for 1 h (*P* < 0.01; Fig. 5c, d). After pretreatment with the JAK1/2 inhibitor—NCB018424, P-STAT1 expression levels were significantly reduced in hPMSCs treated with IL-27 for 1 h (*P* < 0.05; Fig. 5e, f). Moreover, expression levels of PDL1 in hPMSCs incubated with IL-27 for 24 h were significantly decreased when pretreated with the JAK1/2 inhibitor—INCB018424 for 1 h (Fig. 5g, h).

## Discussion

MSCs have become a significant area of research focus in clinical immunology due to their capacity to oppose immunomodulatory effects and their biological characteristics of low immunogenicity. MSCs can regulate a number of biological functions in a variety of immune cells [27–30]. Their inhibitory effects upon T cells are of interests for use in the prevention and treatment of autoimmune diseases [31, 32]. MSCs can be isolated from bone marrow, fat, placenta, skin, and other tissues [11]. We found that placental tissue contains high levels of MSCs, which have similar immunomodulatory effects as those derived from bone marrow, including negative effects upon regulating the activation and proliferation of T cells [19].

IL-27 is predominantly produced from activated macrophages and dendritic cells, which can exert either positive or negative regulatory effects upon immune responses. It has been reported that IL-27 can accelerate the progression of arthritis by promoting Th1 cell generation; however, IL-27 can also alleviate arthritis by suppressing Th17 responses [33]. In addition, IL-27 has also been shown to upregulate the expression of PDL1 in T cells and, in this way, negatively regulate T cell immune responses [6]. Previous results from our laboratory have revealed that hPMSCs inhibit T cell activation and proliferation through stimulating PDL1 expression [21]. Besides, the immunomodulatory effects of hPMSCs could also be affected by cytokine. However, it is unclear whether IL-27 affects immune regulatory effects of hPMSCs. Based upon results obtained from RT-PCR, gene sequencing, Western blot, and FCM assays, we found that IL-27Rα is expressed in hPMSCs; and findings from a related report have indicated that gp130, which is another subunit of the IL-27 receptor, is also expressed in placenta tissue [4]. Taken together, the results of our current experiment suggest that the biological functions of hPMSCs may be affected by IL-27 during immune responses.

In response to inflammation, hPMSCs can migrate to damaged tissue for activation of the immune response, whereas cytokines affect biological functions of hPMSCs. It has been reported that the inflammatory cytokines, IL-1β and TNF-α, can affect the migration of MSCs [34]. However, it is unclear whether IL-27 also affects hPMSC migration. In this study, we first examined whether the adherence, migration, and proliferation of hPMSCs were affected by IL-27 as determined with use of RTCA. With this assay, we found that IL-27 at a concentration of 30 ng/ml significantly inhibited adherence and proliferation, whereas IL-27 at both 20 and 30 ng/ml promoted hPMSC migration. These results suggest that IL-27 can regulate the biological activity of hPMSCs.

Findings from recent studies have indicated that BMSCs can alleviate the symptoms of EAE by regulating Th1 and Th2 responses and promoting IL-10 secretion [14]. However, the immunosuppressive effects of hPMSCs as regulated by IL-27 remain to be determined. In this study, we found that IL-27 can promote the effects of hPMSCs in enhancing the differentiation of PHA-activated T cells into Th2 subsets, IL-10 secretion from CD4^+^T cells, and inhibition of Th1 cell generation by hPMSCs. These results are consistent with previous findings indicating that IFN-γ and TNF-α can regulate the inhibitory effects of hPMSCs on T cell function [17]. In this way, an inverse relationship exists between hPMSCs and inflammatory factors. While hPMSCs can regulate inflammatory factors secreted from immune cells, their immunomodulatory effects are regulated by inflammatory cytokines. Therefore, when applying hPMSCs in the treatment of inflammatory diseases, inflammatory factor levels in patients may affect the clinical outcome of hPMSCs.

PDL1, also known as B7H1/CD274, is an important regulatory molecule involved with inhibiting T cell immune responses. Previous findings from our laboratory have shown that PDL1 plays an important role in the regulation of hPMSCs on T cell responses [21]. Based on that study, we blocked the expression of PDL1 in hPMSCs using PDL1-blocking antibodies to investigate their molecular mechanisms. With this protocol, we found that the capacity for hPMSCs to induce the generation of Th1 or Th2 and IL-10 secretion from CD4^+^T cells was partially reversed, suggesting an involvement of PDL1 in this process. Based on these results, we next examined the effect of IL-27 on the expression of PDL1 in hPMSCs. Our current results demonstrate that IL-27 can upregulate PDL1 expression in hPMSCs. Further work involved with examining the potential molecular mechanisms of these effects has revealed that IL-27-treated hPMSCs have higher expressions of P-STAT1. With the addition of the JAK1/2 inhibitor, expressions of both P-STAT1 and PDL1 in hPMSCs were significantly decreased. Taken together, these findings indicate that IL-27 upregulates PDL1 levels in hPMSCs through the JAK/STAT1 signaling pathway.

In conclusion, our current results demonstrate that (1) IL-27Rα expression is present in hPMSCs, (2) IL-27 exerts inhibitory effects upon hPMSC adherence and proliferation, and (3) IL-27 promotes migration of hPMSCs. In addition, our results also indicate that IL-27 can upregulate PDL1 expression in hPMSCs through the JAK/STAT1 pathway. Such effects enhance the regulatory effects of hPMSCs on Th1 and Th2 cell differentiations and IL-10 secretion from CD4^+^T cells. These findings provide new and important insights into understanding the mechanism of hPMSCs for potential use in clinical cell therapies.
